# Reproductive functions of Kisspeptin/KISS1R Systems in the Periphery

**DOI:** 10.1186/s12958-019-0511-x

**Published:** 2019-08-09

**Authors:** Yubin Cao, Zeping Li, Wenyu Jiang, Yan Ling, Haibin Kuang

**Affiliations:** 1 Department of Physiology, Basic Medical College, Nanchang University, Nanchang, Jiangxi 330006 People’s Republic of China; 2 Department of Clinic medicine, School of Queen Mary, Nanchang University, Nanchang, Jiangxi 330006 People’s Republic of China; 3 Department of Obstetrics and Gynecology, Jiangxi Province People’s Hospital, Nanchang, Jiangxi 330006 People’s Republic of China; 4 Jiangxi Provincial Key Laboratory of Reproductive Physiology and Pathology, Medical Experimental Teaching Center, Nanchang University, Nanchang, Jiangxi 330006 People’s Republic of China

**Keywords:** Kisspeptin, KISS1R, Ovary, Testis, Uterus, Placenta

## Abstract

Kisspeptin and its G protein-coupled receptor KISS1R play key roles in mammalian reproduction due to their involvement in the onset of puberty and control of the hypothalamic-pituitary-gonadal axis. However, recent studies have indicated a potential role of extra-hypothalamic kisspeptin in reproductive function. Here, we summarize recent advances in our understanding of the physiological significance of kisspeptin/KISS1R in the peripheral reproductive system (including the ovary, testis, uterus, and placenta) and the potential role of kisspeptin/KISS1R in reproductive diseases. A comprehensive understanding of the expression, function, and potential molecular mechanisms of kisspeptin/KISS1R in the peripheral reproductive system will contribute to the diagnosis, treatment and prevention of reproductive diseases.

## Introduction

Different species have evolved various survival strategies, but reproduction is an indispensable function of all species permanence. Reproductive function is driven by a complex neuro-hormonal system, with considerable contribution by the hypothalamic-pituitary-gonadal (HPG) axis. The HPG axis is divided into three main levels with the following regulatory signals: 1) hypothalamus: gonadotropin-releasing hormone (GnRH); 2) pituitary: gonadotropin, luteinizing hormone (LH) and follicle-stimulating hormone (FSH); and 3) gonads: sex steroids and peptides [1]. In the regulation of the reproductive system, GnRH neurons are the main hub, and their regulation is complicated, as a wide range of cell types and signalling molecules directly or indirectly converge on the GnRH neuron network [2]. Many regulators of GnRH neurons act through G protein-coupled receptors (GPCRs). KISS1R is one of the most important GPCRs in the neuroendocrine control of reproductive function, and its ligand kisspeptin has a significant effect on the hypothalamus [3]. However, the expression of *KISS1* and *KISS1R* in peripheral reproductive tissues led us to hypothesize that kisspeptin signalling is involved in the local regulation of reproduction within these tissues [4–6]. In particular, three recent reviews have discussed the role of KISS/KISSR signalling in the ovary, the reproductive axis, implantation and placentation [7–9]. In this review, we focus on the local expression and regulation of kisspeptin and its receptor KISS1R in the peripheral reproductive system, including in the ovary, testis, uterus, and placenta, and highlight the potential role of kisspeptin/KISS1R in reproductive diseases.

### The role of kisspeptin in pubertal onset

Kisspeptin is an Arg-Phe-NH_2_ (RF-amide) peptide encoded by the *KISS1* gene [10]. The *KISS1* gene was named after Hershey’s chocolate kisses because it was initially isolated from human non-metastatic pigment tumours in Hershey (Pennsylvania, USA), and the “SS” represents “suppressor sequence” [11]. In humans, the *KISS1* gene is located on chromosome 1q32.11 and encodes a 145-amino acid peptide that is cleaved into four shorter peptides: KP-54, KP-14, KP-13, and KP-10 of 54, 14, 13 and 10 amino acids, respectively. These forms all share a common C-terminal decapeptide (KP-10), which is required for binding with its receptor KISS1R (also known as GPR54) [12]. In humans, kisspeptin is synthesized in two major sections of the hypothalamus: the arcuate nucleus and the anterior ventral periventricular nucleus [13]. The binding of kisspeptin to KISS1R activates the phospholipase C pathway in hypothalamic cells, leading to changes in cellular activity [14]. Current evidence suggests that the kisspeptin signalling pathway is essential for the onset of mammalian puberty. Loss of KISS1R function causes human hypogonadotropic hypogonadism (HH), and one manifestation of HH is the failure to establish puberty due to impaired gonadotropin secretion [15]. The phenotype of human *KISS1R* mutation is mimicked in *Kiss1r* knockout mice [16]. In addition, *Kiss1* knockout rats lack the pulsing and proliferative patterns of gonadotropin and show puberty failure [17]. Conversely, mutations that cause hyperactive KISS1R in humans lead to central precocious puberty [18, 19]. These results suggest that kisspeptin plays an integral role in the regulation of pubertal onset. However, emerging evidence indicates the involvement of extra-hypothalamic kisspeptin and the KISS1R system in peripheral reproductive functions.

### Ovarian kisspeptin and KISS1R

#### Distribution in ovarian tissues

The expression of *Kiss1* and *Kiss1r* was first demonstrated in the rodent ovary [4]. To date, the expression of *Kiss1*/*Kiss1r* has been found in the ovaries of different animals, such as hamsters [20], mice [21], rats [22], chickens [23], cats [24], dogs [25], pigs [26], humans and marmoset primates [27]. Because ovarian *Kiss1* mRNA is mainly expressed in rat granulosa cells during proestrus, granulosa cells are likely the main site of kisspeptin synthesis [28]. The LH surge may directly stimulate kisspeptin synthesis through LH receptors on granulosa cells [29], and prevention of the preovulatory gonadotropin surge can block the upregulation of ovarian *Kiss1* expression [22]. The expression of ovarian *Kiss1* mRNA shows a distinctive cell- and stage-specific pattern under regulation of LH [22, 29, 30], whereas *Kiss1r* mRNA expression remains low and does not significantly fluctuate with the oestrous cycle or gonadotropin treatment in rats [28–30]. Interestingly, in both rodent and human growth follicles, kisspeptin is present in theca cells of the growing follicle; in preovulatory follicles, kisspeptin begins to appear in the basal cells of the granular layer; after ovulation, positive immunostaining can be observed in non-luteinized granulosa cells of newly ruptured ovulation follicles; and in the corpus luteum (CL), intense kisspeptin immunoreactivity can be detected in steroidogenic granulosa lutein cells, with a gradual increase with gradual maturation of the CL [22, 27]. These results demonstrate that kisspeptin and its receptor have a highly conserved expression pattern in rodent, monkey and human ovaries. The distribution of kisspeptin in the ovary has significant temporal and spatial specificity, suggesting that the kisspeptin/KISS1R system performs multiple functions at different physiological stages in the ovary.

#### The role in follicular development

The expression of ovarian *Kiss1* mRNA gradually increases from infancy to adolescence [28]. The immature ovary shows negligible *Kiss1* expression [22], and there is no significant difference in ovarian weight between *Kiss1/Kiss1r*-deficient mice and normal mice before puberty [31]. However, after puberty, the ovaries in Kiss1r^−/−^ and Kiss1^−/−^ mice shrink compared with those in control mice, likely due to the loss of kisspeptin-mediated regulation of follicular development, not defects in gonadotropin secretion because follicular development cannot be rescued by gonadotropin replacement [32]. In fact, although the role of the HPG axis cannot be completely ruled out, follicles at all stages and the CL are present in mice with targeted removal of kisspeptin and Kiss1r neurons (> 90%), suggesting that local kisspeptin in the ovary plays a very important role in follicle development [1].

**Fig. 1:**
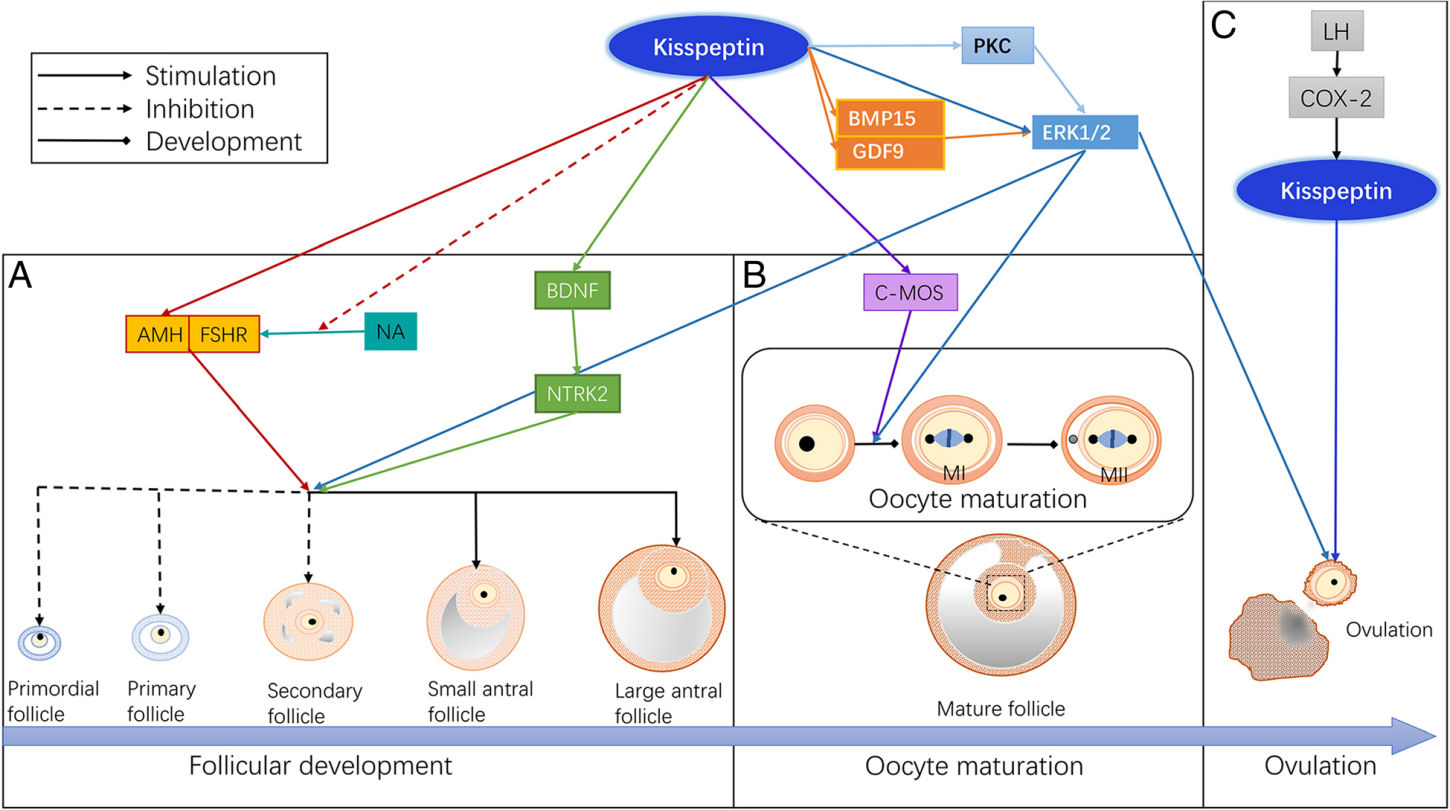
The role of kisspeptin/KISS1R system in the ovary. Ovarian-derived kisspeptin regulates follicular development, oocyte maturation, and ovulation in autocrine or paracrine manner. **a** The possible role and mechanisms of kisspeptin in follicular development. **b** The possible role and mechanisms of kisspeptin in oocyte maturation. **c** The role and mechanisms of kisspeptin in ovulation. AMH, anti-Mullerian hormone; BDNF, brain-derived neurotrophic factor; BMP15, bone morphogenetic protein 15; COX-2, Cyclooxygenase-2; FSH, follicle stimulating hormone; FSHR, follicle stimulating hormone receptor; GDF9, growth differentiation factor 9; LH, luteinizing hormone; NA, noradrenaline; NTRK2, neurotrophic tyrosine kinase receptor type 2; PKC, protein kinase C

Under conditions of a healthy nutrient supply, the administration of kisspeptin in the ovary reduces the number of antral follicles and increases the number of preovulatory follicles, and these structural changes can be reversed by the administration of the kisspeptin antagonist peptide 234 (P234). Furthermore, kisspeptin administration increases plasma anti-Mullerian hormone (AMH) in 6- and 10-month-old rats. AMH, a marker of ovarian reserve, is mainly secreted by secondary and small sinus follicles and can inhibit the activation of primordial follicles by negative feedback; moreover, P234 administration reduces plasma AMH levels in rats [33]. The FSH/follicle stimulating hormone receptor (FSHR) axis is responsible for follicular growth [34], but kisspeptin can block the increase in *FSHR* expression by isoproterenol (ISO, a β-adrenergic agonist). Collectively, kisspeptin negatively regulates the development of preantral follicles by inducing the production of AMH and reduces the sensitivity to FSH by inhibiting the induction of *FSHR* expression by sympathetic activators, thereby reducing the recruitment of primary follicles (Fig. 1a). In the future, an ovarian-specific *Kiss1/Kiss1r* knockout model will be established to further elucidate the role of kisspeptin in follicle development.

#### The role in oocyte maturation

The addition of kisspeptin to FSH-rich medium for porcine cumulus-oocyte complexes (COCs) promotes oocyte maturation, indicating a direct effect of kisspeptin on oocytes [35], and the mechanism may involve upregulating the expression of *C-MOS*, growth differentiation factor 9 (*GDF 9*) and bone morphogenetic protein 15 (*BMP 15*) [36]. Even in the absence of cumulus cells, kisspeptin can increase the maturity of oocytes because *Kiss1r* is expressed in oocytes during in vitro maturation (IVM). Thus, kisspeptin may act continuously and directly on oocytes in an autocrine-paracrine manner. Interestingly, the absence of FSH results in failed oocyte maturation, even in IVM medium supplemented with kisspeptin, confirming a critical role of gonadotropins in the maturation of oocytes in vitro. Moreover, the addition of FSH to COCs induces a significant increase in *Kiss1r* expression, reflecting the permissive action of FSH on kisspeptin.

When a mouse oocyte acquires meiotic capacity, *Kiss1* mRNA expression increases 82.2-fold [36]. However, when the oocyte progresses through the first and second divisions of meiosis (MII), *Kiss1* mRNA expression decreases by 5.4- and 12-fold, respectively [36]. During the progression from germ-vesicle I to MII, the expression of *Kiss1r* remains stable. However, kisspeptin treatment fails to affect the percentage of MII eggs [36]. Therefore, the upregulation of *Kiss1* expression may be related to the ability to undergo meiosis and may affect the recovery of meiosis but not the progression of MII. Taken together, these data suggest that the effect of kisspeptin on oocyte maturation may be accomplished through the regulation of meiosis (Fig. 1b).

#### The role in ovulation

The LH peak plays a crucial role in ovulation by inducing the upregulation of *COX-2* and prostaglandin production [37]. The COX-2 inhibitor NS398 and the COX non-selective inhibitor indomethacin significantly inhibited *Kiss1* mRNA expression in the rat ovary and decreased the efficiency of rat ovulation, suggesting that *Kiss1* may be a downstream target of COX-2 (Fig. 1c). Furthermore, administration of prostaglandin E_2_ can reverse the antagonism of indomethacin on *kiss1* expression. The anti-progestin RU486 ameliorates ovulation defects caused by indomethacin but cannot reverse the regulation of ovarian *Kiss1* expression [27], implying the existence of other pathways that regulate ovulation. In fact, the indispensable role of ovarian kisspeptin in ovulation is suspect because gonadotropins can induce ovulation in *Kiss1*-deficient mice with mild hypogonadism and in women with homozygous *KISS1R* mutations [38].

#### The role in ovarian steroidogenesis

Kisspeptin stimulates progesterone secretion by rat luteal cells and by chicken and porcine granulosa cells. Our previous study showed that recombinant KP-10 significantly enhances basal and human chorionic gonadotropin (hCG)-induced progesterone levels in cultured rat luteal cells and upregulates the transcription of key steroidogenic enzymes (*StAR*, *CYP11A*, and *3β-HSD*) [30]. Moreover, KP-10 promotes the secretion of progesterone by cultured chicken follicular granulosa cells in vitro, accompanied by the upregulation of *StAR*, *CYP11A*, and *3β-HSD* expression [23]. In addition, KP-10 significantly enhances progesterone production and prevents the efflux of oestradiol from granulosa cells of porcine large follicles [23]. Furthermore, KP-10 increases the phosphorylation of the mitogen-activated protein kinase Erk1/2 but not of P38 MAPK and Akt in cultured rat luteal cells, suggesting that kisspeptin may stimulate progesterone secretion via the Erk1/2 signalling pathway in these cells [30]. However, treatment with KP-54 alone did not alter steroidogenesis or the expression of gonadotropin receptors [39], indicating that KP-54 may require gonadotropins to promote steroidogenesis [30] or that different kisspeptin isoforms (such as KP-10) may have different affinities for ovarian KISS1R [23].

Unlike progesterone, KP-10 does not promote the basal or hCG-induced secretion of oestrogen by rat luteal cells [30]. Currently, the best data on the effects of kisspeptin on luteal cell function are from luteinized granulosa cell cultures. KP-54 significantly augments the expression of oestrogen receptors alpha and beta (ESR1 and ESR2) in human granulosa lutein cells, suggesting that kisspeptin may increase sensitivity to oestrogen [39].

Additional studies have indicated that serum kisspeptin levels are significantly higher in women with polycystic ovary syndrome (PCOS), which is characterized by hyperandrogenism and ovulatory dysfunction [40]. Notably, serum levels of kisspeptin are negatively correlated with FSH but positively correlated with LH, testosterone and dehydroepiandrosterone (DHEA) [41]. Mouse KP-10 and KP-52 can significantly increase serum testosterone levels in mice [42]. Furthermore, ovary-derived kisspeptin has been shown to regulate the secretion of LH [43].

### Testicular kisspeptin and KISS1R

#### Distribution in testicular tissues

There are not only significant differences in the distribution of testicular kisspeptin and KISS1R between mammals and non-mammalian species but also diverse distribution patterns in the same or similar species [5, 44–48] (summarized in Table 1). For example, a previous study reported kisspeptin and Kiss1r immunoreactivity in round spermatids in immature mice [5]. However, another study showed kisspeptin immunoreactivity mainly in Leydig cells and sperm cells at different stages, not in only round sperm cells [47]. Therefore, the different results in the same species may be related to the age of the experimental mice and largely influenced by experimental methods. For example, when the *LacZ* gene was inserted into the *Kiss1* and *Kiss1r* alleles to allow β-galactosidase staining to detect gene expression, unique structural changes in sperm (deformation) resulted in inactivation of β-galactosidase after the round spermatid stage, making it impossible to determine whether kisspeptin is expressed in prolonged spermatid and spermatozoa [5].

**Table 1:** The expression of of kisspeptin/KISS1R system in the testis

Reference	kisspeptin	KISS1R	methods	species
Mei et al. [5]	Round SPT	Round SPT, LCs (−)	X-GAL staining and IHC	Mouse
Pinto et al. [45]	SPZ	SPZ	IF, WB	Human
Irfan et al. [46]	Interstitial	SCs	ICC	Monkey
Anjum et al. [47]	Interstitial, LCs, Primordial GCs, elongated GCs, Degenerated GCs	No data	IHC	Mouse
Chianese et al. [44]	Interstitial	Interstitial, PMCs, SCs, ISPG, IISPG, ISPC, IISPC, SPT, SPZ	IHC	Frog
Meccariello et al. [6]	No data	Interstitial, ISPG, IISPG, PMCs	ISH	Frog

SPT, spermatids; LCs, Leydig cells; SPZ, spermatozoa; SCs, Sertoli cells; GCs, germ cells; PMCs, peritubular myoid cells; ISPG, primary spermatogonia; IISPG, secondary spermatogonia; ISPC, primary spermatocytes; IISPC, secondary spermatocytes;

IHC, immunohistochemistry; IF, immunofluorescence; WB, western blot; ICC, Immunocytochemistry; ISH, In situ hybridization

#### The role in spermatogenesis

In non-mammalian species, subcutaneous injection of synthetic Kiss1 pentadecapeptide can speed up spermatogenesis in prepubertal male chub mackerel [47]. In mammals, gene expression profiling revealed that the initiation of *Kiss1/Kiss1r* expression in mouse testis coincides with the formation of spermatozoa [5], suggesting a link between spermatogenesis and the testicular kisspeptin/Kiss1r system in mammals. In addition, kisspeptin exerts anti-metastatic effects by inhibiting cell chemotaxis and migration, which play important roles in the early stage of spermatogenesis [49]. Furthermore, in the late stage of spermatogenesis, KP-13 can induce human sperm motility changes and hyperactivation, possibly caused by the increase in sperm intracellular Ca^2+^ concentration ([Ca^2+^]i) [45]. The positive association between kisspeptin concentration in seminal plasma and semen quality supports the importance of the kisspeptin system in spermatogenesis [50]. However, peripheral kisspeptin may not be essential for spermatogenesis in mammals. First, *Kiss1* and *Kiss1r* mutant mice still show low levels of spermatogenesis on a phytoestrogen diet [51]. Second, male patients with *KISS1R* mutations respond to exogenous hormonal therapy and successfully achieve fertility [52]. Collectively, testicular kisspeptin may not be necessary for mammalian spermatogenesis but is an important regulator of this process.

#### The role in testicular steroidogenesis

Androgens (mainly testosterone) are steroid hormones secreted by Leydig cells in the testes of males. Thus far, there is no verdict as to whether peripheral kisspeptin has an effect on androgen production in Leydig cells. First, the interruption of *Kiss1* expression is associated with decreased testosterone levels in rats [53], and the kisspeptin antagonist P234 reduces the production of hCG-activated testosterone in vitro [54], but local injection of P234 does not alter plasma testosterone levels in adult rhesus monkeys [55]. Second, although the immortalized Leydig cell line MA-10 expresses Kiss1r, it does not respond to KP-10 stimulation [5]. In addition, Sertoli cells respond to kisspeptin and stimulate the production of androgen-binding protein (ABP), indicating a potential role of kisspeptin in ABP production [46].

### Roles in the uterus and placenta

#### The role in the uterus

**Fig. 2:**
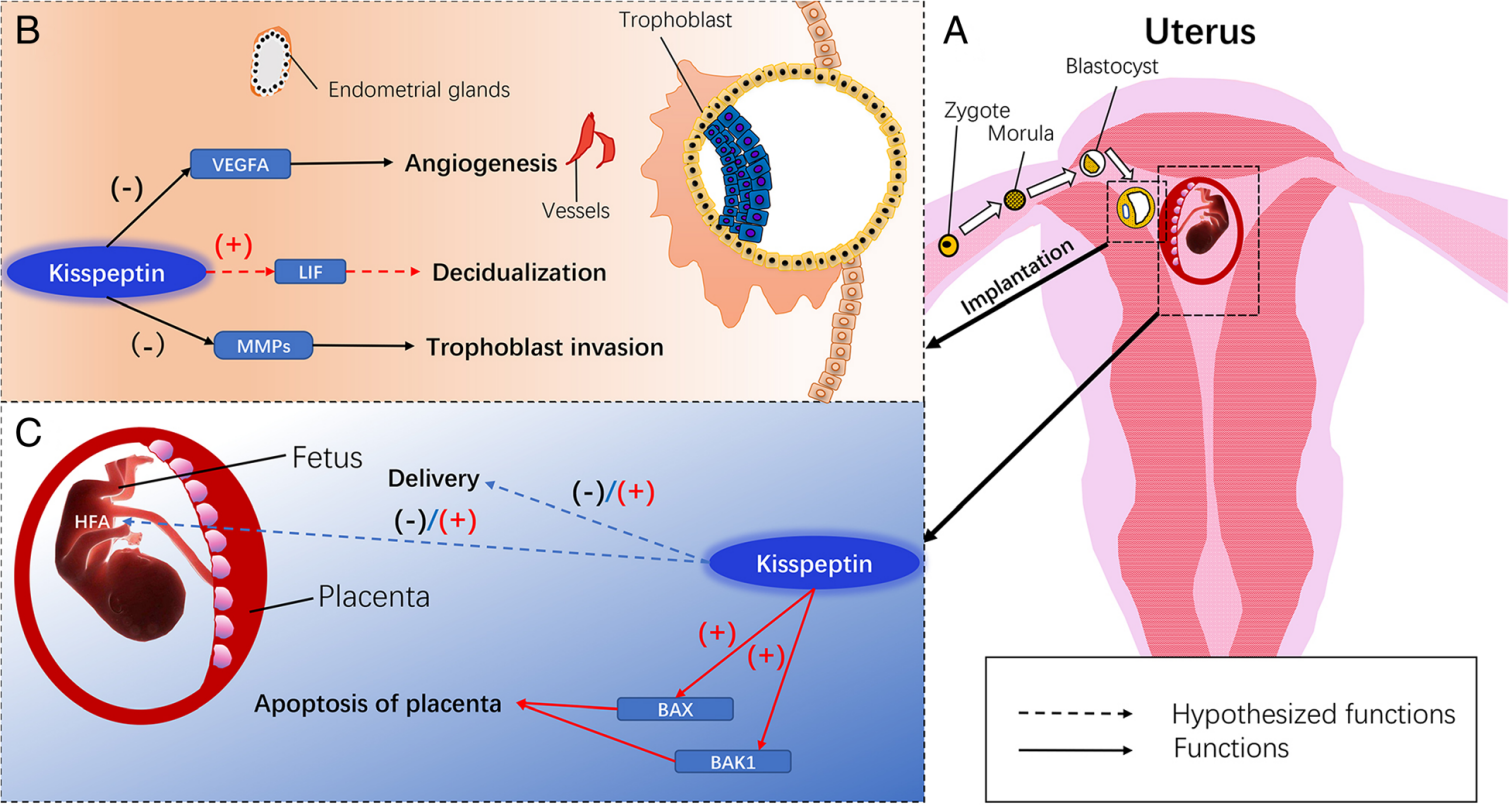
The potential role of kisspeptin/KISS1R system in the pregnancy. **a** A schematic diagram of zygote development, embryo implantation and fetal development in the uterus. **b** The known and potential mechanisms of locally produced kisspeptin in embryo implantation. **c** The known and potential mechanisms of peripheral kisspeptin in fetal development. HFA, human fetal adrenal; MMPs, matrix metalloproteinases; LIF, leukemia inhibitory factor; VEGFA, vascular endothelial growth factor A

In the human female genital tract, KISS1/KISS1R is mainly expressed in epithelial and stromal cells of the uterus but not of the myometrium [6]. In mice, *Kiss1* and *Kiss1r* mRNA expression levels are generally low from day 1 to 4 of pregnancy, which is the stage of zygote to blastocyst transformation (Fig. 2a). KISS1 and KISS1R proteins are mainly localized at low levels in the luminal and glandular epithelium. However, *Kiss1* and *Kiss1r* mRNA expression level dramatically increase with the progression of uterine decidualization, and attenuated expression of *Kiss1* can significantly inhibit the expression of stromal cell decidualization markers, indicating that the kisspeptin/kiss1r system plays an important role in the decidualization process [56]. However, the underlying mechanism is unknown.

Calder et al. found that in kiss1 mutant mice, gonadotropin and oestradiol replacement could restore ovulation, mating, and fertilization but not lead to pregnancy; moreover, leukaemia inhibitory factor (Lif), a crucial cytokine required for implantation, is weakly expressed in these mice [57]. Lif secreted by the uterine glands promotes embryo-uterine communication and contributes to embryo attachment and decidualization [58, 59]. Oestrogen upregulates *Lif* expression in the uterus, and supplementation with Lif restores implantation and decidualization in ovariectomized mice and mice lacking uterine oestrogen receptor expression [60, 61]. Furthermore, in Kiss1 knockout mice, exogenous administration of Lif, but not E_2_, partially rescues implantation failure [57], and our data demonstrated that E_2_ significantly increases the expression of uterine *kiss1* mRNA in ovariectomized mice [56]. These data suggest that kisspeptin signalling may act downstream of E_2_ to stimulate uterine *Lif* expression and is beneficial for promoting embryo implantation and decidualization in mice (Fig. 2b).

#### The role in pregnancy

There is evidence that the primary source of circulating kisspeptin is trophoblast cells of the placenta [12, 62]. In rat placental cells, *Kiss1* expression is upregulated by GnRH and neurokinin B, and all of these neuropeptides can increase hCG expression [63]. Serum KP-54 levels increase several thousand fold during pregnancy and return to normal within 15 days after delivery, suggesting that the placenta produces large quantities of kisspeptin during pregnancy [4, 62, 64]. Moreover, low circulating kisspeptin levels during pregnancy are associated with an increased risk of miscarriage. Therefore, plasma kisspeptin levels are a potential biomarker for miscarriage in the first and third trimesters [65, 66]. As one of the biomarkers of pregnancy, peripheral kisspeptin has multiple functions, including the regulation of placental invasion and migration (discussed in detail below) [62], the apoptosis of embryonic and placental cells, and foetal development [67, 68].

Kisspeptin administration increases the apoptosis of embryonic cells cultured in vitro by upregulating pro-apoptotic genes [69]. The expression of the pro-apoptotic gene *BAK1* in blastocysts increased 3.5-fold at 24 h after kisspeptin treatment, but no significant change was observed in the expression of the anti-apoptotic gene *Bcl-2* [35]. In addition, the apoptosis index (AI), the ratio of the pro-apoptotic protein BAX to the anti-apoptotic protein Bcl-2, determines whether the cell will initiate apoptosis [70]. Interestingly, the AI and *KISS1/KISS1R* expression in the placenta are much higher in late pregnancy than at term delivery in humans [68]. Furthermore, external administration of kisspeptin increases AI and induces apoptosis in placental explants in a dose-dependent manner [68]. Taken together, these data indicate that kisspeptin may be a pro-apoptotic placental factor during pregnancy.

In addition, studies have indicated that the kisspeptin/KISS1R system in the embryo may affect human foetal adrenal function synergistically with adrenocorticotropic hormone and corticotropin-releasing hormone secretion by increasing the production of DHEA in mid to late gestation (Fig. 2c) [71, 72].

#### The role in placental migration and invasion

Kisspeptin was originally called metastin because it can inhibit tumour metastasis. Interestingly, the invasion processes of placental and tumour cells are markedly similar [73, 74]. The highest expression of *Kiss1* and *Kiss1r* in gestational trophoblast cells is consistent with peak trophoblast invasion [62, 75]. Thus, kisspeptin is thought to inhibit trophoblast migration and invasion in the placenta. A series of studies demonstrated that kisspeptin can regulate trophoblast migration and invasion by a variety of mechanisms. First, kisspeptin stimulates Erk1/2 phosphorylation in trophoblast cells and inhibits the expression of matrix metalloproteinases (MMPs), such as *MMP-2*, thereby regulating placental invasion [74, 76]. Second, KP-10 inhibits the migration of HTR8SVneo cells by stimulating complex Erk1/2-GSK3β-FAK feedback interactions in vitro [77]. Third, kisspeptin suppresses angiogenesis by downregulating vascular endothelial growth factor A (Fig. 2b) [78]. In addition, the active kisspeptin/KISS1R system not only suppresses the migration of trophoblast cells but also inhibits their growth in placental explants [35].

## Conclusion

Recently, kisspeptin analogues and KISS1R antagonists have been developed as modulators of the cascade upstream of GnRH, and most previous studies have focused on the central control of the kisspeptin/KISS1R system in the hypothalamus. However, as discussed in this review, the kisspeptin/KISS1R system plays a direct role in peripheral organs (including the ovary, testis, uterus, and placenta) and is implicated in reproductive diseases such as miscarriage and PCOS. A comprehensive understanding of the expression, function, and potential molecular mechanisms of kisspeptin/KISS1R in the peripheral reproductive system will contribute to the diagnosis, treatment and prevention of reproductive diseases.

## Data Availability

All data supporting the conclusion of this article are included in this published article.

## Abbreviations

ABP: Androgen-binding protein
AI: Apoptosis index
AMH: Anti-Mullerian hormone
BMP15: Bone morphogenetic protein 15
CL: Corpus luteum
COCs: Cumulus-oocyte complexes
DHEA: Dehydroepiandrosterone
ESR1: Estrogen receptors alpha
ESR2: Estrogen receptors beta
FSH: Follicle stimulating hormone
FSHR: Follicle stimulating hormone receptor
GDF9: Growth differentiation factor 9
GnRH: Gonadotropin-releasing hormone
GPCRs: G protein-coupled receptors
hCG: Human chorionic gonadotropin
HH: Hypogonadotropic hypogonadism
HPG axis: Hypothalamic-pituitary-gonadal (HPG) axis
IBMX: Isobutylmethylxanthine
ISO: Isoproterenol
IVM: In vitro maturation
LH: Gonadotropin, luteinizing hormone
Lif: Leukemia inhibitory factor
MII: Second division of meiosis
P234: Peptide 234
